# Electrospun Ibuprofen-Loaded Blend PCL/PEO Fibers for Topical Drug Delivery Applications

**DOI:** 10.3390/polym16131934

**Published:** 2024-07-06

**Authors:** Diala Bani Mustafa, Tsuyoshi Sakai, Osamu Sato, Mitsuo Ikebe, Shih-Feng Chou

**Affiliations:** 1 Department of Mechanical Engineering, College of Engineering, The University of Texas at Tyler, Tyler, TX 75799, USA; dbanimustafa@patriots.uttyler.edu; 2 Department of Cellular and Molecular Biology, School of Medicine, The University of Texas Health Science Center at Tyler, Tyler, TX 75708, USA; tsuyoshi.sakai@uttyler.edu (T.S.); osamu.sato@uttyler.edu (O.S.); mitsuo.ikebe@uttyler.edu (M.I.)

**Keywords:** polycaprolactone (PCL), polyethylene oxide (PEO), electrospun fibers, ibuprofen (IBP), mechanical properties, surface wettability, in vitro drug release, in vitro viability assays

## Abstract

Electrospun drug-eluting fibers have demonstrated potentials in topical drug delivery applications, where drug releases can be modulated by polymer fiber compositions. In this study, blend fibers of polycaprolactone (PCL) and polyethylene oxide (PEO) at various compositions were electrospun from 10 wt% of polymer solutions to encapsulate a model drug of ibuprofen (IBP). The results showed that the average polymer solution viscosities determined the electrospinning parameters and the resulting average fiber diameters. Increasing PEO contents in the blend PCL/PEO fibers decreased the average elastic moduli, the average tensile strength, and the average fracture strains, where IBP exhibited a plasticizing effect in the blend PCL/PEO fibers. Increasing PEO contents in the blend PCL/PEO fibers promoted the surface wettability of the fibers. The in vitro release of IBP suggested a transition from a gradual release to a fast release when increasing PEO contents in the blend PCL/PEO fibers up to 120 min. The in vitro viability of blend PCL/PEO fibers using MTT assays showed that the fibers were compatible with MEF-3T3 fibroblasts. In conclusion, our results explained the scientific correlations between the solution properties and the physicomechanical properties of electrospun fibers. These blend PCL/PEO fibers, having the ability to modulate IBP release, are suitable for topical drug delivery applications.

## 1. Introduction

Various types of polymeric drug delivery systems (DDSs) have been developed for drug delivery and tissue engineering applications, such as nanoparticles (e.g., liposomes), microfibers, films, hydrogels, and 3D tissue scaffolds [1]. Among them, drug-eluting microfibers are considered as an excellent candidate for the topical drug delivery of mucosal tissues. These drug-eluting microfibers possess unique physicomechanical properties based on the polymer compositions and the corresponding processing techniques. In addition, their high surface area to volume ratios makes the surface properties more pronounced than the bulk properties in the modulation of drug releases. The high apparent porosities of the fiber meshes allow for the exchange of oxygen and the absorption of physiological fluids. These advantages enable drug-eluting microfibers to be an ideal approach for controlled drug release in wound healing applications.

Several methods have been developed for the fabrication of drug-eluting microfibers, including template synthesis, phase separation, self-assembly, electrospinning and centrifugal spinning [2]. Among these microfiber-making techniques, electrospinning stands out as a robust, cost-effective, and flexible approach to precisely control fiber diameter and morphologies [3]. During electrospinning, micro/nano fibers are drawn from polymer solutions or melts under a strong electric field. As fibers continuously travel across the electric field, solvent evaporates as polymer molecular chains entangle, causing an instability known as “whipping”. Electrospinning parameters, including the applied voltage, flow rate, the distance between the tip of the needle and the grounded collector, polymer solution properties, and environmental conditions all play interconnected roles for the fabrication of continuous microfibers with smooth fiber surfaces. Several works of literature have reported the effects of these processing conditions on the average fiber diameters and fiber morphologies [4,5,6].

Many synthetic polymers have been electrospun into microfibers for drug delivery applications [7]. Hydrophobic polycaprolactone (PCL) and water-soluble polyethylene oxide (PEO) are popular in electrospinning due to their linear and flexible chains as well as their compatibility with many organic solvents. Our previous work on the electrospun 20 wt% ethyl cellulose (EC) and 5 wt% PEO blend microfibers loaded with ibuprofen (IBP) demonstrated the electrospinnability of two vastly different polymers using a common solvent of chloroform, with the ability to modulate fiber physicomechanical properties as well as their in vitro release behaviors [8]. In addition, we electrospun 15 wt% PCL and 4 wt% chitosan (CS) blend microfibers loaded with a hydrophilic model drug (i.e., acetylsalicylic acid, known as Aspirin) in a highly volatile solvent of hexafluoro-2-propanol (HFIP) for PCL and 90% acetic acid solution for CS, to evaluate the effects of blend fiber compositions on the in vitro drug release [9]. Both works demonstrated the importance of fiber surface wettability due to blend fiber compositions on the modulation of drug releases.

Several studies have reported the utilization of PCL/PEO blend fibers as drug carriers. For instance, surface modified PCL/PEO fibers were prepared in dimethylformamide/chloroform (10/90) solvent, using 18 wt% PCL and 1 wt% PEO, for the encapsulation of curcumin [10]. An in vitro release study showed a burst release of curcumin in the first hour followed by a steady release up to 67% at 48 h. Others electrospun various compositions of blend PCL/PEO fibers using 12 wt% of the polymers in dichloromethane/dimethylformamide (80/20) solvent to load doxycycline up to 21% (*w*/*w*) loading [11]. The results showed that PCL/PEO (0/100) and (100/0) fibers exhibited 100% and 65% releases of doxycycline in 120 min, whereas other blend fibers showed release profiles in between the two formulations. Additionally, using the PCL/PEO (75/25) fibers, increasing doxycycline loading promoted the burst release to about 95% within the first 120 min. In another study, 15 wt% PCL/PEO fibers were prepared in chloroform, followed by the loading of ibuprofen (IBP) for electrospinning [12]. The in vitro release of IBP from blend PCL/PEO fibers reached 85% and 96% at 8 and 72 h, respectively. These findings highlighted the potential use of PCL/PEO blend fibers for controlled drug delivery in biomedical applications.

In this study, we extend our previous works to explore the electrospinning of two synthetic polymers (i.e., PCL and PEO) using a common solvent (i.e., chloroform) with a model drug, IBP. Our goal is to produce blend fibers using a fixed polymer concentration, to better correlate the physicomechanical properties with the in vitro drug release behaviors. To achieve this goal, the hypothesis is that increasing the PEO composition in the blend PCL/PEO fibers promotes the surface wettability of the fiber meshes, resulting in a faster release of IBP. Our results showed that microfibers were only electrospinnable at PCL/PEO compositions of 75/25, 50/50, and 25/75 when using 10 wt% blend polymer solutions in chloroform. We showed that the average fiber diameters depended on the blend fiber compositions, which further affected the average fiber mechanical properties. In addition, fiber wettability remained as the critical factor to modulate the in vitro release of IBP. Using the standard cell culture procedure followed by the MTT assay, IBP-loaded blend fibers at various compositions were compatible to MEF-3T3 fibroblasts. Our results demonstrate the scientific importance of solution viscosities on the physicomechanical properties of electrospun blend fibers, as well as their potential engineering applications in drug release, suitable for topical dressings in wound healing applications.

## 2. Materials and Methods

### 2.1. Materials

Polycaprolactone (PCL), with an average molecular weight (M_w_) of 80,000 Da, was purchased from Huaian Ruanke Co., Ltd. (Huaian City, China). Polyethylene oxide (PEO), POLYOX^TM^ WSR 205, with an average molecular weight (M_w_) of 600,000 Da, was kindly supplied by DuPont de Nemours, Inc. (Wilmington, DE, USA). Ibuprofen (IBP), 98% purity, was supplied by Ark Pharm, Inc. (Arlington Height, IL, USA). Chloroform (>99.8% stabilized ACS), dimethyl sulfoxide (DMSO, ≥99.9% ACS), and biotechnology grade phosphate-buffered saline (PBS) buffer solution (pH~7.3–7.5) were purchased from Avantor (Radnor, PA, USA). Other chemicals were of reagent grade from the suppliers for the study, without further purification.

### 2.2. Preparation of Polymer Solutions

Various volumetric compositions of blend PCL/PEO solutions (e.g., 100/0, 75/25, 50/50, 25/75, and 0/100) were prepared by dissolving predetermined amounts of the polymers in chloroform. Briefly, masses of PCL beads and PEO powders were measured using a Mettler Toledo AG245 analytical balance (Columbus, OH, USA), followed by dispensing chloroform into glass vials to achieve 10% (*w*/*v*) of PCL/PEO solutions. Glass vials containing PCL/PEO in chloroform were placed on a Thermo Scientific^TM^ Labquake^TM^ rotisserie mixer (Waltham, MA, USA) for dissolution at room temperature overnight. Similarly, the procedures were repeated for the preparation of a 30% PCL/PEO (100/0) solution and an 8% PCL/PEO (0/100) solution.

For the preparations of the IBP-loaded PCL/PEO solutions, predetermined amounts of IBP were measured using a Mettler Toledo AG245 analytical balance (Columbus, OH, USA) prior to adding them to the corresponding blend PCL/PEO solutions at 15% (*w*/*w*) loading.

### 2.3. Viscosity Measurements

Viscosities were measured using the falling ball method (Stokes’ law), where 3 mL of each blend polymer solution was drawn into the syringe and placed on the mount to ensure the filled syringe is stable for viscosity measurements. A digital camera was placed in front of the syringe to record the time for the steel ball to fall through a known distance of 39.3 mm in the polymer solution. The steel ball had a diameter of 6.35 mm and a weight of 1.044 g, and the viscosity results were averaged on five independent measurements (*n* = 5).

### 2.4. Fiber Electrospinning

All polymer solutions were visually examined with care for undissolved particles before electrospinning. Polymer solutions were placed into a 3-mL BD Luer-lok^TM^ disposable syringe (Franklin Lakes, NJ, USA), attached with a 21-gauge needle. A NE-1000 programmable single syringe pump (Farmingdale, NY, USA) was used to dispense the polymer solutions from the syringe-needle assembly. The syringe pump was calibrated for the 3-mL BD syringe to dispense polymer solution at a flow rate of 25 μL/min. During electrospinning, a total of 3 mL of the polymer solution was dispensed, using an applied voltage of 12.5 kV with a deposition distance of 15 cm. Fibers were collected on a grounded stationary collector plate that was covered in a layer of wax paper. After electrospinning, fiber meshes were covered with another layer of wax paper to prevent contamination prior to storing them in a vacuum desiccator.

### 2.5. Fiber Morphologies and Average Fiber Diameter Measurements

The morphologies of the electrospun blank and IBP-loaded PCL/PEO blend fibers were evaluated using a scanning electron microscope (SEM). Circular disk punches of the fiber mesh were placed on the carbon tape for imaging. SEM images were captured using a Hitachi TM4000Plus System (Tokyo, Japan) at 15 kV, with a working distance of approximately 5.5 mm.

Average fiber diameters were measured using the ImageJ software (version 1.54i), National Institutes of Health (Bethesda, MD, USA), on the collected SEM images. Specifically, 50 random measurements were taken from SEM micrographs to determine the average fiber diameter and the standard deviation of each sample (*n* = 50).

### 2.6. Thermochemical Characterizations

Thermal analyses of blank and IBP-loaded PCL/PEO blend fibers were performed using a Shimadzu DSC-60 Plus (Kyoto, Japan) at a scanning rate of 20 °C/min between 25 and 120 °C, according to a previous study [13]. The weight of the specimens ranged between 8 and 10 mg.

The chemical structures of blank and IBP-loaded PCL/PEO blend fibers were measured using a Thermo Scientific™ Nicolet Avatar 360 FTIR system (Walthem, MA, USA), according to the previously published method [13]. The spectra were recorded between 4000 and 400 cm^−1^ with a resolution of 8 cm^−1^. Peak information from various spectra were analyzed using OMNIC™ software (version 8.1.11).

### 2.7. Mechanical Testing

Uniaxial tensile tests were carried out using a single column screw driven Instron^®^ 3342, universal materials testing equipment (Norwood, MA, USA), equipped with a 100 N load cell. ASTM standard dog-bone specimens (22 mm nominal length and 5 mm width) were prepared using an ODC stainless steel die (Waterloo, ON, Canada) [14]. The nominal thickness of each dog-bone sample was measured by a digital thickness gauge (resolution = 10 μm). Uniaxial tensile tests were performed in accordance with ASTM standard D5034-21, using a strain rate of 0.01/s [15]. Load and displacement data were recorded from the instrument to calculate stress and strain. A total of 3 specimens were used for each blend fiber composition (*n* = 3).

### 2.8. Surface Wettability Measurements

A number of 3/8″ circular discs were taken from each blend fiber formulation for water contact angle measurements. Each fiber disc was placed on a metal substrate for the deposition of a 4 μL PBS droplet at the center of the fiber disc. A digital camera was placed in front of the fiber discs to record the water contact angle when the droplet was just in contact with the fibers. The average water contact angles were analyzed using ImageJ software (version 1.54i), National Institutes of Health (Bethesda, MD, USA), from three independent samples (*n* = 3).

### 2.9. In Vitro Drug Release Studies

A number of 3/4″ circular discs were taken from each of the IBP-loaded PCL/PEO blend fibers for in vitro drug release studies. Phosphate-buffered saline (PBS) buffer solution (pH~7.3–7.5) was used as the release media, due to its similarity to the physiological condition of human tissue. The masses of the fiber discs were measured to determine the theoretical drug loadings and the corresponding PBS volumes to ensure a sink release condition. Glass vials, containing predetermined amounts of PBS, were placed into a Thermo Scientific^TM^ MaxQ 4450 orbital shaker (Waltham, MA, USA) to prewarm to 37 °C, prior to placing the IBP-loaded fiber disc samples into each corresponding vial for shaking at 120 rpm.

A 40 µL liquid sample, containing an unknown concentration of the IBP released from the fibers, was removed from each glass vial and placed in a microcentrifuge tube using a clean pipette tip for each extraction at predetermined time points of 5, 10, 15, 30, 60, and 120 min. After each extraction of the liquid sample from the glass vials, a 40 µL of fresh PBS was pipetted into each glass vial to ensure consistent volume of the total release media.

IBP standard solutions were prepared in PBS/DMSO (1/1), using the serial dilution method from 400 μg/mL to 25 μg/mL. Both the standard IBP solutions and the unknown IBP liquid specimens collected at various time points were analyzed using a Thermo Scientific^TM^ NanoDrop^TM^ 1000 UV–vis spectrophotometer (Waltham, MA, USA) at 265 nm. The resulting UV–vis intensities of IBP, from the unknown samples at various released time points, were compared to the standard IBP curves and the theoretical loading of the IBP to determine the in vitro cumulative release of IBP. The results were average on three independent measurements (*n* = 3).

### 2.10. In Vitro Cell Biocompatibility

Mouse embryonic fibroblast 3T3 cells (MEF-3T3), obtained from the American Type Culture Collection (ATCC) (Manassas, VA, USA), were cultured in fresh culture media of Dulbecco’s modified Eagle medium (DMEM), supplemented with 10% fetal bovine serum (FBS) and 1% antibiotic mixture. The cell cultures were incubated in a humidified chamber with 5% CO_2_ at 37 °C, followed by sub-culturing the cells in 96-well plate at 50% confluency (approximately 1 × 10^4^ cells/well) prior to the viability assays.

The MEF-3T3 viability assays were carried out in accordance with previous studies [16]. Briefly, tissue culture polystyrene (TCPS) plate control groups were supplemented with standard culture media, whereas the IBP control groups included mixtures of 10% and 1% dilutions of the saturated IBP solutions (i.e., 21 μg/mL [17]) with the standard culture media, respectively. For the experimental groups, the in vitro release media, collected from various IBP-loaded PCL/PEO fiber vials after 48 h, were diluted to 10% and 1% in culture media prior to MTT assays. After the incubation of the cells with the MTT reagent, a 100 μL warm detergent solution was then added to each well to lyse the cells and solubilize the colored crystals for the collection of the supernatants. The supernatants were then transferred to a 96-well plate for optical measurements using a TECAN Infinite^®^ 200 Pro M Plex microplate reader (Männedorf, Switzerland) at 590 nm absorbance. All experiments were performed in triplicate (*n* = 3), and the results were expressed as relative cell viability ratio compared to the control groups.

### 2.11. Statistical Analyses

The results were expressed as average ± standard deviation (SD). Statistical studies of the averages were performed using GraphPad Prism (San Diego, CA, USA) on one-way analysis of variance (ANOVA). Significance was accepted with *p* < 0.05.

## 3. Results

### 3.1. Blend PCL/PEO Solution Properties

**Figure 1:**
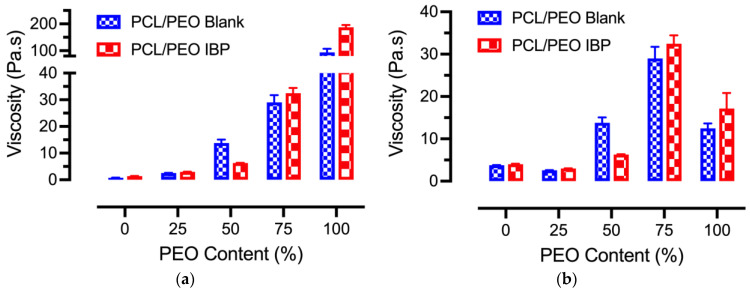
Average viscosities of the blank and IBP-loaded PCL/PEO solutions: (**a**) 10 wt% polymer concentrations for all PCL/PEO blends; (**b**) 30 wt% polymer concentrations for PCL/PEO (100/0) blends, 8 wt% polymer concentration for PCL/PEO (0/100) blends, and 10 wt% polymer concentrations for the remaining PCL/PEO blends, using the same average viscosity data as in (**a**) for plotting.

The average viscosities of various 10 wt% blank and IBP-loaded PCL/PEO solutions are shown in Figure 1a. The average viscosities of the blank and IBP-loaded PCL/PEO solutions showed exponential increases from 0.75 ± 0.05 Pa.s to 93.50 ± 13.56 Pa.s and from 1.36 ± 0.05 Pa.s to 187.37 ± 8.71 Pa.s, respectively, with the inclusion of PEO due to its high molecular weight. In addition, the incorporation of the small molecule drug, IBP, at 15% (*w*/*w*) loading in various PCL/PEO solutions, showed higher average viscosities at all PCL/PEO formulations, except the 50/50. It has been reported that polymer solutions with a viscosity between 0.1 to 2 Pa.s and a surface tension between 3.5 to 5.5 N/m^2^ are suitable for electrospinning [18]. While the viscosity and the surface tension on the electrospinnability of the polymer solutions depend on the polymer–solvent system, our results showed that both the blank and IBP-loaded PCL/PEO formulations at 100/0 and 0/100 were either too thin or too thick for electrospinning.

In order to electrospin the blank and IBP-loaded PCL/PEO formulations at 100/0 and 0/100 in chloroform, polymer concentrations of 100/0 and 0/100 formulations were adjusted to 30 wt% and 8 wt%, respectively. The average viscosities of the blank and IBP-loaded PCL/PEO solutions, after adjusting the polymer concentrations, were 3.77 ± 0.05 Pa.s and 3.99 ± 0.11 Pa.s for the 100/0 formulation and 12.48 ± 1.15 Pa.s and 17.17 ± 3.66 Pa.s for the 0/100 formulation, respectively. Figure 1b shows the superimposed average viscosity data with the adjusted average viscosities of 100/0 and 0/100 formulations, which suggests a viscosity range of 3 to 30 Pa.s to electrospin PCL/PEO solution in chloroform. The data reported from the following sections were in accordance with the viscosity data from Figure 1b.

### 3.2. Fiber Electrospinning

The blend PCL/PEO polymer solutions, 100/0 (30 wt%), 75/25 (10 wt%), 50/50 (10 wt%), 25/75 (10 wt%), and 0/100 (8 wt%), were electrospun into fibers. In order to standardize the electrospinning parameters for all the formulations of various polymer concentrations, a series of tests were performed to evaluate the minimal voltage and the maximum flowrate needed at a 15 cm collector distance. The purpose of these tests was to set a standard electrospinning voltage and flowrate for all formulations to inform jet formation.

Table 1 shows the minimal voltage needed and maximum flowrate possible for the electrospinning of various blank and IBP-loaded PCL/PEO formulations. For minimal voltage setup, the applied voltage increased when increasing PEO concentration in the PCL/PEO blend solutions. The IBP-loaded PCL/PEO solutions appeared to have a lowered applied voltage as compared to the blank solutions. For the maximum flowrate setup, the flowrate increased slightly when increasing PEO concentration in the PCL/PEO blend solutions. The IBP-loaded PCL/PEO solutions demonstrated higher flowrates than the blank counterparts. Based on the results from electrospinning study, various concentrations of PCL/PEO solutions were electrospun into fibers using 12.5 kV, with a flowrate of 25 μL/min under the collector distance of 15 cm.

### 3.3. Fiber Morphologies and Average Fiber Diameters

**Figure 2:**
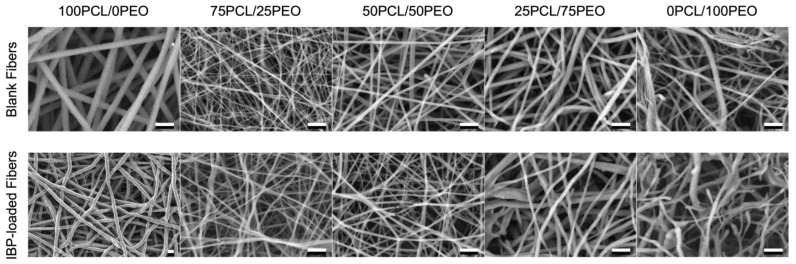
SEM images of electrospun blank and IBP-loaded PCL/PEO blend fibers using various volumetric compositions. The 100PCL/0PEO and 0PCL/100PEO fibers were electrospun using 30 wt% and 8 wt% solutions, respectively. Others were produced with 10 wt% solutions. Scale bar = 10 μm.

The surface morphologies of the blank and IBP-loaded PCL/PEO blend fibers were investigated using SEM. Figure 2 displays the SEM images of the electrospun PCL/PEO blend fibers at various compositions. All the fiber formulations showed smooth surfaces with uniform fiber diameter. These fibers were defect-free (e.g., beads and webbings), suggesting the effective correlations between the solution properties and electrospinning parameters.

**Figure 3:**
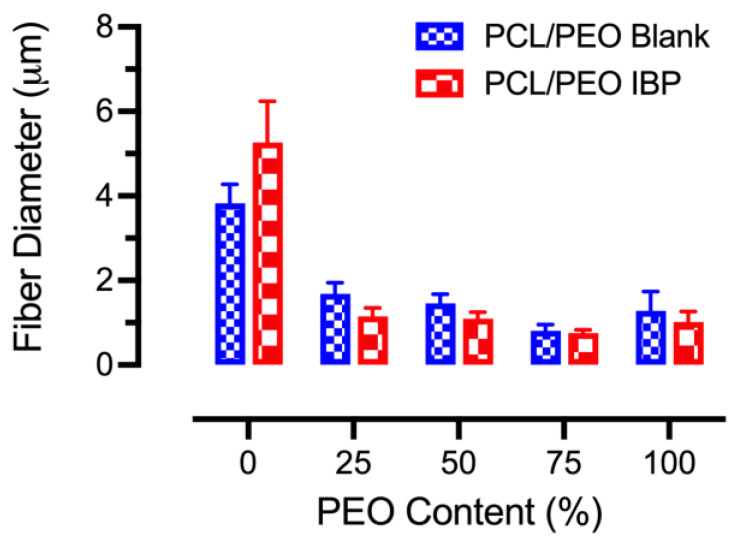
Average fiber diameters for electrospun blank and IBP-loaded PCL/PEO fibers using virous polymer compositions. The PCL/PEO (100/0) and PCL/PEO (0/100) fibers were electrospun with 30 wt% and 8 wt% solutions, respectively. Others were produced with 10 wt% solutions.

The average fiber diameters of the blank and IBP-loaded PCL/PEO blend fibers at various concentrations are shown in Figure 3. The average fiber diameters for the 30% PCL/PEO (100/0) fibers were 3.83 ± 0.44 μm and 5.26 ± 0.98 μm for the blank and IBP-loaded fibers, respectively. In contrast, the average fiber diameters for the 8% PCL/PEO (0/100) fibers were 1.28 ± 0.45 μm and 1.02 ± 0.25 μm for the blank and IBP-loaded fibers, respectively. The average fiber diameters of the 10% blend PCL/PEO fibers (i.e., 75/25, 50/50, and 25/75) decreased with increasing PEO contents. The incorporation of the IBP in the 10% blend PCL/PEO fibers slightly decreased the average fiber diameters as compared to their counterparts, except in the 100/0 formulation.

### 3.4. Thermochemical Characterizations

**Figure 4:**
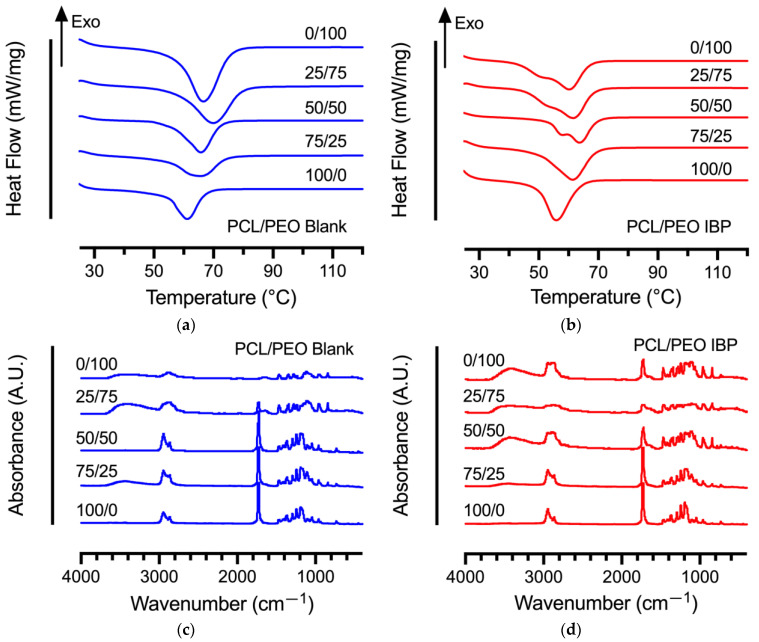
Differential scanning thermograms and Fourier transform infrared spectra of electrospun PCL/PEO blend fibers at various compositions: (**a**) DSC thermograms of blank PCL/PEO fibers; (**b**) DSC thermograms of IBP-loaded PCL/PEO fibers; (**c**) FTIR spectra of blank PCL/PEO fibers; (**d**) FTIR spectra of IBP-loaded PCL/PEO fibers.

DSC experiments were performed to identify possible drug-polymer interactions in various blank and IBP-loaded PCL/PEO fibers. Figure 4a,b show the representative DSC thermograms of the blank and IBP-loaded PCL/PEO fibers at various compositions. The melting temperatures of PCL and PEO are 56 °C and 63 °C, respectively [19]. Incorporating PEO in the blend PCL/PEO fibers increased the melting temperatures of the blend fibers due to the higher melting temperature of the PEO. Furthermore, the incorporation of the IBP in the blend PCL/PEO fibers decreased the melting temperatures for all fiber compositions as compared to their blank counterparts, suggesting the plasticizing effect of IBP in blend PCL/PEO fibers, similar to the reported studies [20,21].

The FTIR spectra of the blank and IBP-loaded PCL/PEO fibers are shown in Figure 4c,d. The characteristic peaks of PCL included the stretching vibrations of the ester carbonyl groups (–C=O) at 1727 cm^−1^, symmetric and asymmetric stretching vibrations of the ester linkages (C–O–C) in the carbon backbone at 1100 cm^−1^ and 1150 cm^−1^, and the symmetric and asymmetric stretching of –C–H at 2883 cm^−1^ and 2943 cm^−1^, respectively [16]. The FTIR characteristic peaks of PEO consisted of doublet at 963 cm^−1^ and 947 cm^−1^, associated with the rocking vibration of the CH_2_ conformation groups, as well as the symmetric and asymmetric stretching vibrations of C–O–C at 1100 cm^−1^ and 1150 cm^−1^ [19]. The FTIR characteristic peaks of IBP included a sharp peak at 1706 cm^−1^, corresponding to the carboxyl acid groups (COOH), and other smaller peaks between 1000 cm^−1^ and 1200 cm^−1^ from the benzene ring [22]. The broad bands at 3461 cm^−1^ in the IBP-loaded PCL/PEO fibers were associated with the characteristic stretching vibration of the hydroxyl groups (OH) from the inter- and/or intra-molecular hydrogen bonds associated with IBP plasticization.

### 3.5. Mechanical Properties

**Figure 5:**
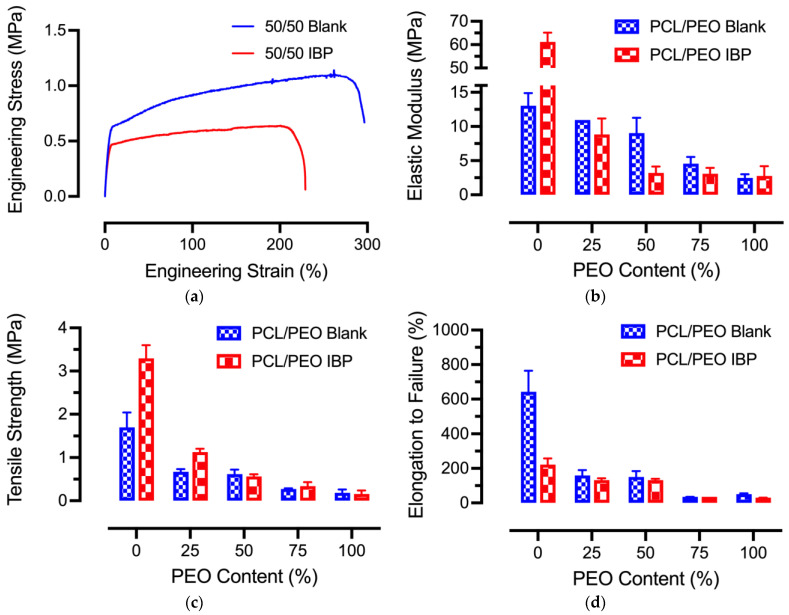
Mechanical properties of electrospun PCL/PEO blend fibers at various compositions: (**a**) Representative stress–strain curves for blank and IBP-loaded PCL/PEO (50/50) fibers; (**b**) Average elastic moduli; (**c**) Average tensile strength; (**d**) Average elongation to failure for the various blend PCL/PEO fibers.

Uniaxial tensile tests were performed on the electrospun blank and IBP-loaded PCL/PEO fibers, and the representative stress–strain curves as well as the mechanical properties are shown in Figure 5. The representative stress–strain curves using PCL/PEO 50/50 fibers, shown in Figure 5a, demonstrated an initial viscoelastic region at about 2% strain, followed by a plastic deformation of the blend polymer fibers. Noticeably, the IBP-loaded PCL/PEO fibers displayed a minimal strain hardening effect in the plastic region, suggesting the role of the plasticization of IBP in PCL/PEO fibers.

The average elastic moduli of the electrospun blank and IBP-loaded PCL/PEO fibers at various compositions are shown in Figure 5b. The average elastic moduli for the 30% PCL/PEO (100/0) fibers were 13.05 ± 1.82 MPa and 61.17 ± 3.98 MPa for the blank and IBP-loaded fibers, respectively. Increasing PEO content gradually decreased the average elastic moduli for the blank and IBP-loaded fibers. In addition, the IBP-loaded PCL/PEO fibers appeared to exhibit lower average elastic moduli than the blank PCL/PEO fibers (except the PCL/PEO 100/0 composition), suggesting the plasticizing effect of IBP in the blend PCL/PEO fiber matrix.

The average tensile strength of the electrospun blank and IBP-loaded PCL/PEO fibers at various compositions, shown in Figure 5c, exhibited similar trends to the average elastic moduli. Increasing PEO content in the blank and IBP-loaded PCL/PEO fibers decreased the average tensile strength. The incorporation of the IBP in the PCL/PEO fibers decreased the average tensile strength as compared to their counterparts. Furthermore, the average elongation to failure, shown in Figure 5d, also decreased when increasing PEO content in the blank and IBP-loaded PCL/PEO fibers. IBP facilitated the fracture of the blend PCL/PEO fibers at smaller strains than their counterparts.

### 3.6. Surface Wettability

**Figure 6:**
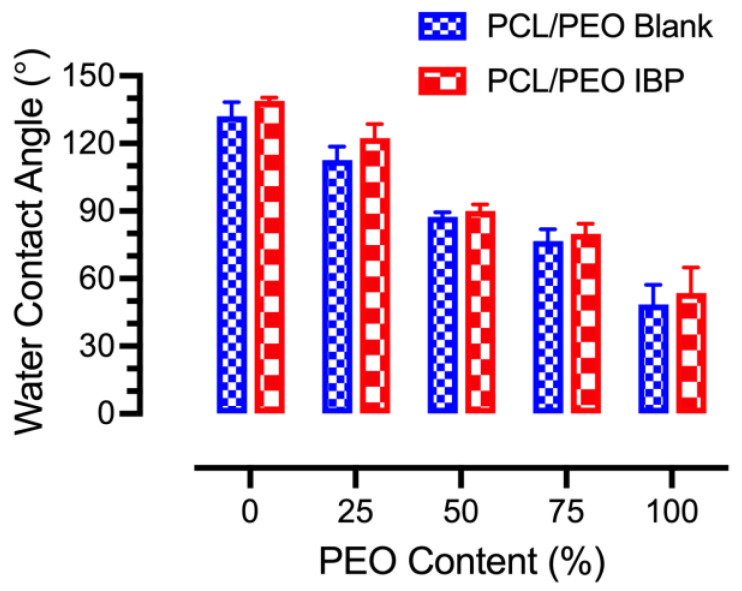
Average water contact angles of blank and IBP-loaded PCL/PEO fibers at various PEO compositions.

Surface wettability of the blank and IBP-loaded PCL/PEO fibers were measured using the water contact angle method, and the results are shown in Figure 6. The average water contact angles of the blank PCL/PEO fibers decreased from 132.14° ± 6.12° to 48.57° ± 8.65° when increasing the PEO content. In addition, the average water contact angles of the IBP-loaded PCL/PEO fibers decreased from 138.95° ± 1.38° to 53.61° ± 11.36° when increasing the PEO content. The decrease in the average water contact angles in various compositions of the blend PCL/PEO fibers was associated with the incorporation of water-soluble PEO. Moreover, incorporating IBP in the PEL/PEO blend fibers enhanced the surface hydrophobicity, due to the lipophilic nature of the IBP.

### 3.7. In Vitro Drug Release Assay

**Figure 7:**
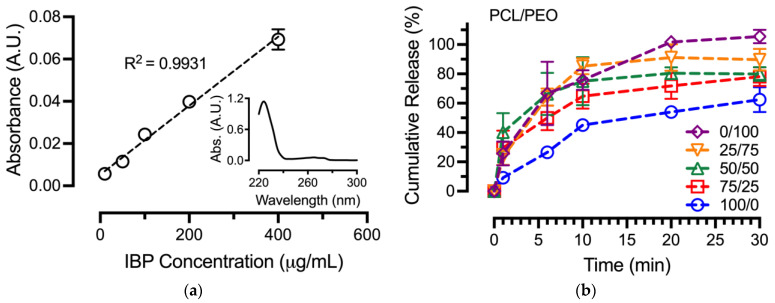
In vitro release of IBP from various compositions of PCL/PEO fibers: (**a**) Standard curve of IBP using PBS/DMSO (1/1) solvent with figure inset showing a representative UV–vis spectrum of IBP; (**b**) Cumulative release of IBP from various PCL/PEO fibers.

The in vitro release of IBP from blend PCL/PEO fibers in the sink condition was performed to correlate various fiber formulations on IBP releases, and the results are shown in Figure 6. Prior to setting up the in vitro release assay, an IBP standard curve was established in a PBS/DMSO (1/1) mixture using the serial dilution method, as shown in Figure 7a. The use of a PBS/DMSO mixture as the solvent was due to the limited solubility of IBP crystals in PBS and/or DI-water. The R^2^ correlation from the linear regression was 0.9931 for the IBP standard curve. In addition, the Figure 7a inset shows a representative UV–vis spectrum of IBP in PBS/DMSO (1/1), with characteristic peaks at 225 nm and 265 nm associated with π → π* transition of the aromatic ring in IBP’s molecular structure [23].

The in vitro release of various electrospun IBP-loaded PCL/PEO fibers was performed in the sink condition, using PBS as the media. During electrospinning, the carboxylic acid groups of the IBP deprotonated (COO^−^) in the neutral condition of chloroform [24]. The resulting ionic IBP compounds that were encapsulated in the blend PCL/PEO fibers became soluble in PBS and/or DI-water during the in vitro release assay. Using the established IBP standard curve, unknown concentrations of IBP at various time points were plotted into cumulative release curves for the in vitro release of IBP from the blend PCL/PEO fibers, as shown in Figure 7b. According to the cumulative release curves, the PCL/PEO 100/0 fibers exhibited an IBP release of 45.1 ± 3.6% after 10 min, followed by a slow release up to 120 min. In contrast, the PCL/PEO 0/100 fibers demonstrated a burst IBP release of 75.8 ± 6.6% after 10 min, suggesting the effects of the surface wettability of electrospun drug-eluting fibers on drug release. The 10 wt% blend PCL/PEO fibers in between the two pure polymers showed a gradual transition from slow to fast release as the composition of PEO increased in the blend fibers. Finally, the excessive cumulative release of the PCL/PEO 0/100 fibers was associated with the drug encapsulations that determined the theoretical IBP loading.

### 3.8. In Vitro Cell Biocompatibility

**Figure 8:**
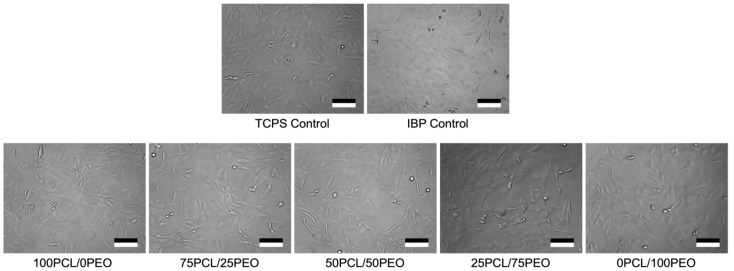
MEF-3T3 cellular morphologies after 48 h of culture in various media, including tissue culture polystyrene (TCPS) plate controls with standard cell culture media, IBP controls with 10% dilution of saturated IBP in standard cell culture media, and various experimental groups of 10% dilution of the release media from PCL/PEO blend fibers in standard cell culture media. Scale bar = 100 μm.

The in vitro cell biocompatibilities of various IBP-loaded PCL/PEO fibers were performed in standard cell cultures of MEF-3T3 using MTT assays. The control groups consisted of standard tissue culture polystyrene (TCPS) plates and the IBP drug crystals in PBS as positive controls. The experimental groups included the in vitro release media from various IBP-loaded PCL/PEO fibers after 48 h of incubation, followed by 10% and 1% dilutions with standard cell culture media for cell cultures. As shown in Figure 8, all MEF-3T3 cells displayed the spindle- and stellate-shaped morphologies for typical fibroblasts after 48 h of culture in 10% dilution of IBP, and the corresponding release media from blend PCL/PEO fibers. Cytoplasmic fragmentation, condensation, and/or shrinkage, which are characteristics of apoptosis, were not observed in the culture, suggesting the in vitro compatibility of IBP-loaded PCL/PEO fibers with fibroblasts.

**Figure 9:**
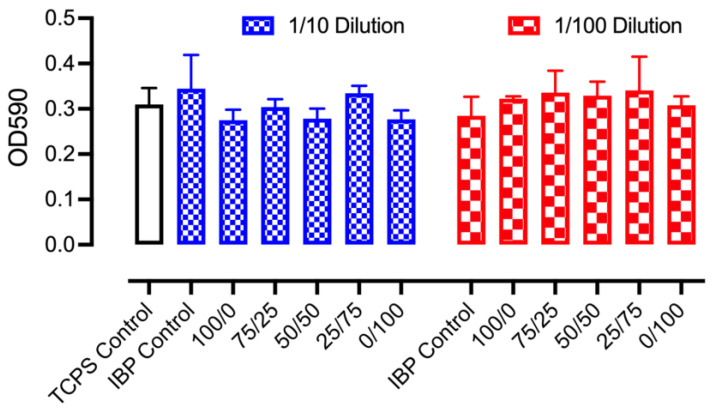
Quantitative cell viability measurements using MTT assays on MEF-3T3 cultures with control groups and various IBP-loaded PCL/PEO fiber groups using mixtures of 10% and 1% fiber release media with the standard culture media.

Figure 9 shows the quantitative analysis of metabolic activities from MEF-3T3 cultured with the release media of IBP-loaded PCL/PEO fibers. The UV absorption of the formazan-forming crystals at 590 nm, indicating the mitochondrial function of the living cells, demonstrated minimal cytotoxicity levels after 48 h of culture with various IBP-loaded PCL/PEO fibers as compared to the TCPS and IBP control groups. As described in the in vitro release section, the culture media consisted of various concentrations of ionic IBP compounds from the positive control and the various fiber groups. Despite the concentrations, the permeation of IBP ionic compounds through the lipid cell membranes became difficult, resulting in minimal cellular uptakes. Consequently, the electrospun IBP-loaded PCL/PEO fibers were compatible with MEF-3T3 after 48 h.

## 4. Discussion

### 4.1. Effects of Solution Viscosities on Fiber Electrospinning

Fiber electrospinning is a uniaxial stretching process on the charged polymer jet, and this process is greatly affected by the viscosities of polymer solutions [25]. It has been found that at very low solution viscosity, electrospinning becomes electrospraying, yielding the production of micro/nano particles instead of continuous fibers [26]. Our findings from both the 10 wt% blank and IBP-loaded PCL/PEO (100/0) solutions were in accordance with the literature, where micro particles were the result of the low average solution viscosities. As such, we intentionally increased the polymer concentration for the PCL/PEO (100/0) solutions to 30 wt% to enable fiber electrospinning. In addition, the competition between the applied voltage and the surface tension of the polymer solution generates the Taylor cone at the tip of the needle, within which polymer chains entangle, leading to jet instability in electrospinning [27]. This effect applied to all the blank and IBP-loaded PCL/PEO solutions of (75/25), (50/50), and (25/75), and therefore, the polymer concentrations for these three formulations were kept at 10 wt%. Increasing the viscosity of the polymer solution increases chain entanglement to a critical viscosity value, where the applied voltage becomes unable to overcome the surface tension of the polymer solution for jet formation [28]. This effect took place with our 10 wt% blank and IBP-loaded PCL/PEO (0/100) solutions, and therefore, the PCL/PEO (0/100) formulations were adjusted to an 8 wt% polymer concentration.

The correlations between the average viscosities of PCL/PEO solutions (Figure 1) and their electrospinning parameters (Table 1) showed that increasing PEO composition in blend PCL/PEO solutions increased the minimal applied voltage and the maximal flow rate for fiber electrospinning, without the spraying and/or dripping of polymer solution from the tip of the needle. The former effect demonstrated that a higher average solution viscosity led to a stronger surface tension of the polymer solution, resulting in an increase of the minimal required voltage in order to electrospin fibers [29]. The latter effect suggested that jet instability was more pronounced in PEO than in PCL, so more fibers were produced at a fixed applied electric field [30]. Jet instability was produced by chain entanglement. Since both PCL and PEO are both linear polymers, the level of chain entanglement depends on the polymer molecular weight (e.g., chain length).

### 4.2. Effects of Average Fiber Diameters on Fiber Mechanical Properties

The average fiber diameters for the blank and IBP-loaded PCL/PEO (100/0) fibers were the highest among all formulations, due to large amounts of polymers/drugs in the solutions [31]. The remaining PCL/PEO formulations had low polymer concentrations, resulting in smaller average fiber diameters. The average fiber diameter of the electrospun fibers had significant implications on the fiber mechanical properties. Studies showed that decreasing average fiber diameters improved the elastic modulus and tensile strength while lowering the fracture strain [32]. This correlation was in contrast to our findings, where smaller average fiber diameters of the blend PCL/PEO (75/25), (50/50), and (25/75) fibers received lower average elastic moduli, average tensile strength, and average fracture stain than the (100/0) and (0/100) formulations. It has been reported that the electrospun PCL fibers (M_w_: 80,000 Da) had an average elastic modulus of 23.7 ± 3.6 MPa, an average tensile strength of 4.7 ± 0.8 MPa, and an average fracture strain of 625 ± 69% [33]. In addition, the electrospun PEO fibers (M_w_: 900,000 Da) showed an average elastic modulus of 6.9 ± 1.4 MPa, an average tensile strength of 1.0 ± 0.1 MPa, and an average fracture strain of 233 ± 30% [8]. The intrinsically different mechanical behaviors of PCL and PEO fibers were the major determining factor in our findings, whereas the blend PCL/PEO fibers followed the rule of mixture.

The incorporation of IBP influenced the mechanical properties of the PCL/PEO blend fibers, compared to their blank counterparts. The decrease in average stiffness and average tensile strength suggested the plasticizing effect of IBP in PCL/PEO blend fibers, supported by the thermochemical analysis in Figure 4. Studies showed that the IBP molecules interacted with ester-based linear PLA during fiber stretching by reducing the intermolecular interactions of the polymer chains to improve chain mobility [34]. Others demonstrated that the average elastic moduli decreased with the incorporation of IBP in PCL/PEO blend fibers, attributed to an enhanced chain mobility that reduced the rigidity of the polymer network [35]. Our studies showed a decrease in average tensile strength of the PCL/PEO blend fibers (except the 75/25 formulation), similar to the reported literature. The extent of the changes in the fiber’s mechanical properties depended on IBP loading and the overall polymer network [36].

### 4.3. Effects of Surface Wettability on Drug Release

Surface wettability is one of the important factors for electrospun fibers in topical drug delivery applications. A water-soluble polymer (e.g., PEO) typically shows a water contact angle below 90°. In contrast, hydrophobic polymers, such as PCL, possess water contact angles above 90° at the surface. Our results were consistent with the literature, where the PCL/PEO blend fibers showed average water contact angles that decreased with increasing PEO content [37]. It was noted that the IBP-loaded PCL/PEO blend fibers had higher average water contact angles than their blank counterparts. These findings were in agreement with our previous study, showing that lipophilic IBP played a significant role in the non-wetting behavior of the electrospun fibers [8].

The in vitro releases of drugs were greatly controlled by the types of polymer matrix. While water-soluble PEO prompted drug release due to polymer dissolution, hydrophobic PCL provided a slower drug release, associated with the non-wetting behavior of the polymer. The blending of water-soluble PEO with hydrophobic PCL offered a viable way to modulate drug release for topical drug delivery [38]. As such, the IBP releases from various PCL/PEO blend fibers in this study were modulated by fiber compositions. Additionally, studies showed that IBP interacted differently at the molecular level with PCL/PEO blend fibers (e.g., drug partition) [12]. Drug–polymer molecular interactions influenced PEO dissolution and the diffusion of IBP from the PCL in our blend fibers. Our results aligned with the hypothesis that blending a water-soluble polymer of PEO with a hydrophobic polymer of PCL modulate the in vitro release of IBP.

## 5. Conclusions

In conclusion, we electrospun blend PCL/PEO fibers at various compositions with 15% (*w*/*w*) IBP loading, using a common solvent of chloroform. We showed that the average polymer solution viscosities of various PCL/PEO blends directly affected the electrospinning parameters for the production of smooth and defect free fibers. The mechanical properties of the blend PCL/PEO fibers exhibited dependance on the average fiber diameter, per the same polymer concentrations. IBP appeared to plasticize the PCL/PEO network to reduce the average elastic moduli, tensile strength, and fracture strain of the fibers. In addition, surface wettability studies correlated with the in vitro release of IBP, suggesting the role of blend polymers in the modulation of drug release through polymer dissolution and drug diffusion. Overall, our study provided a systematic approach to understand the effects of electrospun PCL/PEO blend fibers on the physicomechanical properties, with a focus to identify the roles of blend fibers in drug delivery.

## Figures and Tables

**Table 1:** Electrospinning parameters for various blend PCL/PEO solutions to obtain fibers. The PCL/PEO (100/0) and PCL/PEO (0/100) formulations were utilizing 30 wt% and 8 wt% solutions, respectively, while others were electrospun with 10 wt% solutions. The collector distance was set to 15 cm.

Fiber Formulations (PCL/PEO)	Minimal Voltage (kV) @ 20 μL/min		Maximum Flowrate (μL/min) @ 12 kV	
	Blank	IBP-Loaded	Blank	IBP-Loaded
100/0	7.5	8.0	50	80
75/25	8.5	8.5	65	85
50/50	10.0	8.5	65	85
25/75	10.5	8.5	65	85
0/100	11.0	9.0	70	90

## Data Availability

The data presented in this study are available on request from the corresponding author. The data are not publicly available due to privacy reason.
